# Morphokinetic Characteristics and Developmental Potential of In Vitro Cultured Embryos from Natural Cycles in Patients with Poor Ovarian Response

**DOI:** 10.1155/2016/4286528

**Published:** 2016-12-20

**Authors:** N. Hojnik, V. Vlaisavljević, B. Kovačič

**Affiliations:** ^1^Department of Reproductive Medicine and Gynecologic Endocrinology, University Medical Centre, Maribor, Slovenia; ^2^Biomedical Research Institute (BRIS), Ljubljana, Slovenia

## Abstract

*Background.* Patients with poor ovarian response to ovarian hyperstimulation represent an interesting group for studying the impact of embryo cleavage irregularities on clinical outcome since all embryos, regardless of their quality, are usually transferred to the uterus. The aim of our study was to follow the morphokinetics of fertilized oocytes from natural cycles in poor responders.* Methods.* Zygotes from 53 cycles were cultured in vitro for 3 days. The morphokinetics of their development and transfer outcomes were retrospectively analyzed for the normally and irregularly cleaved embryos.* Results.* Of all embryos, 30.2% had single and 20.8% multiple cleavage irregularities with the following prevalence: developmental arrest 30.2%, direct cleavage to more than two cells 24.5%, chaotic cleavage 13.2%, and reverse cleavage 11.3%. These embryos had longer pronuclear phases, first cytokinesis, second embryo cell cycles, and less synchronized divisions. The transfer of normally developing embryos resulted in an implantation rate of 30.8% and a delivery rate of 23.1%, but irregularly cleaved embryos did not implant.* Conclusions.* The use of time-lapse microscopy in poor responder patients identified embryos with cleavage abnormalities that are related with no or extremely low implantation potential. Gained information about embryo quality is important for counselling patients about their expectations.

## 1. Introduction

Poor ovarian response (POR) to controlled ovarian hyperstimulation presents one of the greatest challenges in reproductive medicine. These patients face a decline in the number and quality of oocytes, which dramatically lowers their chances to conceive. It is estimated that poor response to ovarian stimulation occurs in 9–24% of women [1]. The proportion of poor responders among the population of patients that seek in vitro fertilization (IVF) to conceive is getting higher, with the postponing of parenthood towards the biological age limits.

Despite many studies, a preferred method of ovarian stimulation is still not confirmed for this group of patients. Since the definition of “poor response” was very broad [2], the data on efficacy and success rates of different protocols were confusing. Only recently, with the introduction of the Bologna criteria [3], does there seem to be a better basis for studies that compare the efficacies of natural cycle IVF, modified natural cycle IVF, and various stimulation protocols. There are data in the literature that suggest that it is reasonable to use natural cycle as a method of choice for this group to achieve acceptable results [4]. Increasing the dosages of gonadotrophins does not improve the number of mature oocytes obtained, fertilization rate, or clinical pregnancy rates [5]. There is also some evidence that milder ovarian stimulation reduces the incidence of aneuploidy compared with high dosage stimulation [6]. One of the arguments for treating these patients with natural cycle IVF includes the questionable cost benefits of IVF treatment with high dosages of gonadotrophins that yield small numbers of oocytes [7]. The treatment options also depend on the availability of an oocyte donation program and local legislation and reimbursement policies. Nevertheless, the success rates of natural cycle IVF in women with poor ovarian response according to the Bologna criteria are low, with 2.6% live birth rate per cycle [8].

Poor ovarian response is associated with many factors that lower the success rate. The possible causes for the condition include the woman's age and other factors that affect the pool of follicles during its formation in fetal development and later in her life. It can be the result of ovarian surgery, chemotherapy, radiotherapy, chronic smoking, autoimmune disorders, and various genetic risk factors like X chromosome derangements [9] and fragile X mental retardation 1 (FMR1) premutation [10]. The result is the decline in number and quality of the oocytes. It has been established that miscarriage rate is higher in poor responders comparing with normal responders [11]. In patients with POR, the incidence of aneuploid blastocysts is higher, with 35.1% of all aneuploid blastocyst cycles reported [12]. The prevalence and etiology of the higher ratio of aneuploid embryos in patients with POR are not entirely clear. Poor ovarian response is obviously associated with age and aneuploidy, mainly because of the premature segregation of sister chromatids as a result of depletion of the cohesion [13]. Nevertheless, it is not only a woman's age that affects the ratio of aneuploidy. Trisomic pregnancy is more frequent in women with a reduced ovarian follicle pool, independent of their age [14]. The data of Sunkara et al. also show that not only age but also other factors that cause the decline of oocyte quality in this group of patients affect the miscarriage rate [15]. It is not only the missegregation of genetic material that comes along with aging oocytes; there are also changes in gene expression through DNA methylation, histone modifications, miRNA expression, and mitochondrial function as reviewed by Ge et al. [16].

All these alterations can affect embryo development. With the time-lapse microscopy, we can observe events in early embryo development in vitro. It is established that cleavage abnormalities are quite frequent [17]. They indicate cell anomalies on different levels and they may also give rise to postzygotic genetic abnormalities, such as mosaicism, which arise during mitotic division. There seem to be many different factors associated with these abnormal cleavage patterns, but there is a lack of data regarding what the causes are for certain types. The correlation between aneuploidy and kinetic markers was studied by Campbell et al. [18]. There are also sperm-derived factors that influence embryo cleavage. The centrosome that organizes cleavages is inherited paternally. In an experiment using the rhesus macaque monkey model, the association between sperm that had been exposed to oxidative stress prior to intracytoplasmic sperm injection (ICSI) and the occurrence of cleavage abnormalities was established [19]. The integrity of the spindle in subsequent mitotic events is also very important and its abnormalities contribute to missegregation of the genetic material [20].

The aim of our study was to follow the cleavage patterns and morphokinetics of embryos from natural ICSI cycles in the group of poor responder patients. These patients represent an interesting group for studying the impact of embryo cleavage irregularities on further development and clinical outcome, as every cleaved zygote, regardless of its quality, was transferred into the uterus.

## 2. Materials and Methods

### 2.1. Study Design

Fifty-three consecutive natural cycles with embryo transfer in the period from February 2012 until April 2014 were included in the study. All forty-six patients were poor responders according to the Bologna criteria [3]. Detailed analyses of the morphokinetic parameters of 53 embryos that were transferred into the uterus were performed. Cycles were retrospectively split into those with normally cleaved embryos and cycles with one or more embryo cleavage irregularities. Transfer outcomes (implantation and live birth rate) were compared between both groups. The study was approved by the Institutional board for Medical Ethics.

### 2.2. Patients and Interventions

Patients were diagnosed as poor responders according to the Bologna criteria [3]. In short, they fulfilled two of the following criteria: (i) advanced maternal age or any other risk factor for POR; (ii) a previous POR; and (iii) an abnormal ovarian reserve test (anti-Müllerian hormone [AMH] 0,5–1,1 ng/mL; antral follicle count <5–7). The criteria were also fulfilled in case of two episodes of POR after maximal stimulation, in the absence of advanced maternal age or abnormal ovarian reserve tests. Patients of advanced age with an abnormal ovarian reserve were also included, although they had no previous stimulation.

Patients were treated with ICSI on single oocyte obtained from natural cycle as described previously [21]. In short, they had no downregulation of the pituitary gland or ovarian stimulation and their natural menstrual cycle was followed with ultrasound scans of follicle growth and estradiol measurements. When the follicle was >15 mm and the estradiol was >0.5 nmol/L, 5,000 IU human chorionic gonadotropin (hCG) was administered to trigger ovulation. An ultrasound-guided ovarian puncture with follicle flushing was performed 35 hours later. Embryo transfer was performed on day 3 of cultivation. The luteal phase was supported by administration of 2500 IU of hCG on the day of transfer [22]. Serological pregnancy was tested for 16 days later and was positive if serum *β*-hCG was more than 10 IU/mL. Clinical pregnancy was defined as a presence of gestational sac with positive fetal heartbeat detected at week 6 of pregnancy by transvaginal ultrasound. Live birth rate was defined as the birth of a child.

### 2.3. ICSI, Embryo Culture, and Embryo Transfer

Oocytes were fertilized with ICSI. Correct fertilization was confirmed 16–20 hours afterward when confirming presence of two pronuclei. After the ICSI, the oocytes were immediately transferred into a Primo Vision micro well group culture dish (Vitolife, Göteburg, Sweden) with a 60 *μ*L droplet with preequilibrated sequential medium. The media used for cultivation was ISM 1™ (Origio, Mäløv, Denmark). Cultivation was in the standard humidified incubator with 6% CO_2_ and 5% O_2_ atmosphere at 37°C. Embryos were transferred to the uterus three days after fertilization.

### 2.4. Time-Lapse Microscopy of Early Embryo Development

Embryos were monitored with Primo Vision™ time-lapse embryo monitoring system (Cryoinnovation, Vitrolife, Göteburg, Sweden), which is composed of a microscope unit that is inserted into a classic incubator. Photographs were generated every 5 minutes on 7 focal planes; illumination was green led light, duration 20–30 ms. The development of 53 embryos was analyzed. Embryo development was analyzed retrospectively by observation of the sequence of events that were described according to the proposed guidelines [23]. For each embryo, we described the time elapsed from insemination by ICSI to the extrusion of the second polar body (*t*PB2), the appearance of both pronuclei (*t*PNa), the disappearance of the pronuclei (*t*PNf), and the time period in which the pronuclei were visible (VP). Cell stages were described as the time from ICSI to the first frame in which the membranes of the two, three, or four blastomeres were completely separated (*t*_2_, *t*_3_, *t*_4_). We calculated the duration of the first cell cycle (ECC1 = *t*_2_  −  *t*PB2), the second embryo cell cycle (ECC2 = *t*_4_ − *t*_2_), the synchronization of the cell divisions between the 2- and 4-cell stages (s2 = *t*_4_ − *t*_2_), and duration of the first cytokinesis (dck1), which is the time from the first frame in which the cleavage furrow is visible to the time point at which cytokinesis is completed.

Normal cleavage was defined on the basis of data from time-lapse microscopy as cleavage of the zygote to two blastomeres and subsequent cleavages of each blastomere to two blastomeres.

We defined abnormal developmental patterns according to known data from the literature. The pregnancy rates for direct cleavage in more than two blastomeres are described by Athayde Wirka et al. [17], for reverse cleavage by Liu et al. [24]. The occurrence of irregularities in early embryo development is also described elsewhere [25].

**Figure 1:**
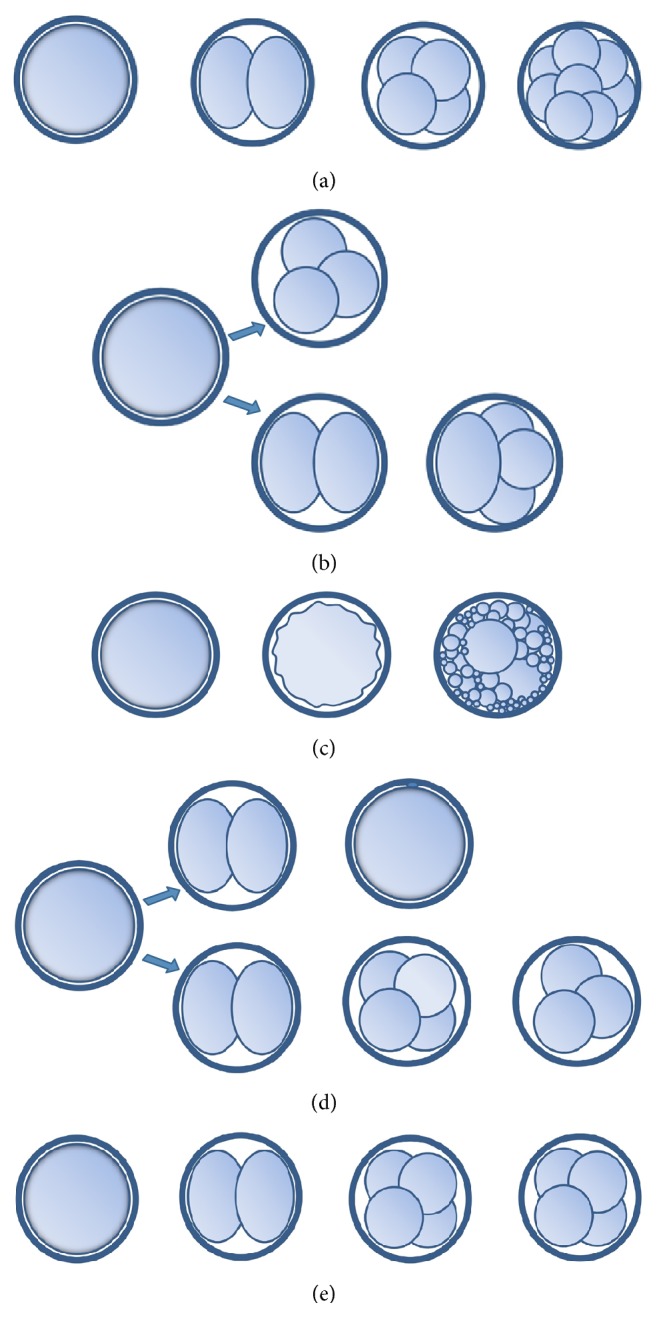
Observed patterns of early embryo development: (a) normal development with division of zygote in two blastomeres in the first cell cycle (ECC1) and second embryo cell cycle (ECC2) leading to 4-cell embryo that can be observed on day 2 of cultivation. In the third embryo cell cycle (ECC3), the 4-cell embryo duplicates all blastomeres and an 8-cell embryo can be observed on day 3 of cultivation. (b–e) Cleavage irregularities: (b) direct cleavage of a cell in more than two blastomeres (in the ECC1 in the upper row and in ECC2 in the lower row) usually with tripolar cleavage furrow that leads to 3 equally sized cells. (c) Chaotic cleavage in ECC1 with no bipolar cleavage furrow observed, membrane ruffling of the zygote, and extensive fragmentation during division in two or more cells. (d) Reverse cleavage: the reduction of the number of blastomeres of an embryo due to blastomere fusion. The process can be observed at any point of development, here presented as reverse cleavage of two blastomeres into one cell. (e) Arrested development: when embryos had fewer blastomeres than expected and did not approximately double the number of cells every 24 hours.

Abnormal cleavage was defined as one or a combination of described types of cleavage irregularities (Figure 1). The first type was direct cleavage of one cell in more than two blastomeres, with the predominant type being direct division in three blastomeres of equal size in the first cell cycle. The second was chaotic cleavage with membrane ruffling, multiple cleavage furrows, division into unevenly sized blastomeres, and large proportion of fragments. The third was reverse cleavage described as blastomere fusion or failed cytokinesis. The fourth was arrested embryo development, defined as embryos that had significantly fewer blastomeres than expected and did not approximately double in number of cells every 24 hours. The expected number of blastomeres is 2–4 on day two of cultivation and 6–8 on day three of cultivation.

Classic morphology was evaluated only once, at the time of the transfer of the embryo to the uterus. We used Istanbul consensus criteria for the cleavage-stage embryos [26]. Morphologically optimal embryos were those with stage-specific cell size (8-cell stage at 68 ± 1 hours after insemination), with less than 10% fragments and no multinucleation. The proportion of morphologically optimal embryos was calculated for each group of embryos (Table 2).

On the basis of data from the time-lapse microscopy, we compared cycles with normally cleaved embryos and cycles with one or more cleavage irregularities. We compared timings of the events, positive hCG, ongoing pregnancies, and live birth rates between both groups.

### 2.5. Statistical Analysis

A statistical analysis was performed using Statistica software (StatSoft, Inc., Dell, Tulsa, USA). Proportion parameters were compared by using Fisher's exact test. Mean values of baseline patient characteristics were compared using the Mann–Whitney *U* test. The timing of the events was compared with Student's *t*-test. *P* < 0.05 was considered statistically significant.

## 3. Results

### 3.1. Patients' Characteristics

In 46 patients, 53 cycles were included in the final analysis of morphokinetic parameters of transferred embryos. The mean age of patients in our study was 39.1 ± 3.0 years. Mean value for AMH was 0.35 ± 0.36. Mean body mass index was 22.9 ± 3,4. Mean number of previous treatment cycles was 2.85 ± 2.06, ranging from no previous cycle to 9 previous cycles, with both stimulated and natural cycles included. Indication for treatment was female infertility in 47.2% of cycles and combined male and female infertility in 52.8% of cycles.

### 3.2. Morphokinetic Parameters and Primary Outcomes

When analyzing morphokinetic parameters, a high incidence of abnormal events in embryo development was established. In 50.9% of cycles, the transferred embryos showed single or multiple cleavage irregularities described above. We compared patient characteristics and outcomes for the two groups of cycles, those with normally developing embryos and those where one or more cleavage irregularities occurred. Regarding the baseline characteristics, women were equally distributed between the two groups. Age, AMH values, body mass index, ratio of cycles with combined male infertility, and estradiol values were comparable. There was a statistically significant difference in the number of previous treatment cycles in that the number was higher in the group of cycles with normally developing embryos.

In the group of normally developing embryos, the implantation rate was 30.8% and the delivery rate per cycle was 23.1%. None of the embryos in the group with one or multiple cleavage irregularities implanted and the difference was statistically significant. Comparison of the baseline characteristics and primary outcomes for the two groups is summarized in Table 1.

The prevalence of abnormal cleavage patterns in embryo development is summarized in Table 2. 50.9% of the embryos had irregularities in early development (27/53). Among all monitored embryos, 30.2% (16/53) had single and 20.8% (11/53) had multiple irregularities detected in early development.

In 34.0% of cycles, the classical morphology score at the time of transfer to the uterus was good quality embryo, while in 38.9% it was fair and in 27.1% poor, according to the Istanbul consensus scoring system. All except two of the embryos with a good quality score were from the group of normally developing embryos and all pregnancies derived from these embryos. Only two morphologically good quality embryos with single cleavage irregularity were observed, but they did not result in pregnancies. In the group of embryos with multiple irregularities, there were no good quality embryos.

The prevalence of four different types of irregularities among 53 monitored embryos was analyzed, taking into account that several different abnormalities can be observed in one embryo. Altogether, 42 irregularities were observed: developmental arrest 16/53 (30.2%), direct cleavage in more than two blastomeres, predominantly in three blastomeres of equal size 13/53 (24,5%), chaotic cleavage 7/53 (13.2%), and reverse cleavage 6/53 (11.3%).

Comparison of the timing of the events in the two groups is summarized in Table 3. No statistically significant differences were found in the time from ICSI to the extrusion of the second polar body, the time from ICSI to the appearance of the pronuclei, the time from ICSI to the fading of the pronuclei, the times from ICSI to the first frames in which 2- and 3-cell stage were observed, and the duration of the first cell cycle. Statistically significant differences were calculated for the time of the duration of the pronuclei, the duration of the first cytokinesis, the duration of the second cell cycle, and the synchronization of the cell division between the 3- and 4-cell stages. Embryos with cleavage irregularities showed statistically significantly longer duration of the pronuclei, longer duration of the first cytokinesis, longer second cell cycle, and less synchronized divisions.

## 4. Discussion

Time-lapse technology in human IVF programs is mainly used as a tool for the selection of the most potent embryo for transfer to the uterus among a cohort of more available embryos in one stimulated cycle. There are many publications focused on finding new morphokinetic parameters with the highest predictive value for implantation [27, 28]. As morphologically optimal embryos are usually selected for transfer among the cohort, implantation and delivery rates are known for them. But it is less known how morphokinetic markers can predict the primary outcomes of a cycle with single embryo where embryo selection is not possible.

In the case of poor responder patients, the criteria for what is still a suitable embryo for transfer are not defined yet. Our data show that a classic morphological score can reflect cleavage irregularities, but not in the case of direct cleavage in more than two blastomeres. Implanted embryos in this study cleaved normally and they all had good morphology scores at the time of transfer. Abnormal divisions did not always result in poor embryo morphology. Direct division in three cells, especially, resulted in the development of more blastomeres, and fragmentation is usually rare in this type of irregular division. None of the embryos with multiple cleavage abnormalities had good morphology scores.

In 50.9% of all cycles, we transferred embryos with one of the major cleavage abnormalities detected with time-lapse microscopy, mainly in the first cell cycle. Early abnormal cleavage has a greater impact on embryo viability than any other irregularity that occurs later, because it affects a greater part of the embryo. The repairing mechanisms enable embryos to repair certain abnormalities. If an aberrant division occurs later, it can be compensated for, because the blastomeres are still pluripotent [29]. But if abnormalities happen early in development, in the first or second embryo cell cycle, these embryos have very little or no repairing potential [17].

In patients with poor ovarian response the cancellation of embryo transfer due to low quality of available embryos in our center is low, since they have difficulties accepting cancellation and we transfer all embryos, regardless of their quality. The implantation rates thus cannot be directly compared with normal responders, where we have the possibility for selection of the best embryo for embryo transfer. The reduced pregnancy rate in poor responder patients seems to be related to the frequent transfer of embryos with cleavage abnormalities that would normally be deselected if more embryos are available.

We analyzed developmental abnormalities detected with the use of time-lapse microscopy in single embryo derived from oocytes obtained from natural cycles. With the conventional method of embryo morphology assessment once per day these abnormalities are usually overlooked. Data about implantation potential of embryos are mainly focused only on the influence of embryo stage and morphological score on the day of embryo transfer on clinical results [30, 31]. In our study, when transferring normally developing embryo, results are relatively good for this group of patients, with 30.8% implantation rate per embryo transferred and 23.1% delivery rate per cycle, regardless of the mean age of women. Moreover, the mean age of pregnant women in our study is 40.4 ± 1.2 years. The implantation and delivery rate in this group are somehow comparable with the general success rate of cycles with elective embryo transfer. Concerning natural cycle in poor responders, it is possible that stringency of the criteria around what to transfer can also contribute to the diversity of the primary outcome results in the literature.

A relatively high incidence of abnormalities in early embryo development was found in this group of patients. The overall prevalence of embryos exhibiting one or more abnormal events was 50.9%. Comparing our data with the data from the literature is difficult since other studies focused on different abnormalities and irregular cleavage patterns than our study and different populations of patients were studied. In the study of Athayde Wirka et al., 54.2% of all embryos derived from stimulated cycles exhibited one or more atypical phenotypes, but they also studied other features such as abnormal syngamy and abnormal first cytokinesis [17]. Prevalence of abnormal divisions in embryos derived from immature oocytes in stimulated cycles was 72.1% [25]. It would be very interesting to find out how the quality of oocytes affects its cleavage pattern.

There is a possible correlation between the stability and competence of genetic material and subsequent divisions. In early human embryogenesis, in the first few mitotic divisions, chromosome missegregation is very common [32]. Mitotic errors contribute significantly to the ratio of cleavage-stage embryo aneuploidy [33]. There is some evidence in the literature that structurally abnormal chromosomes can be one of the causes for disruption of the mitotic process [34]. But there are conflicting results of different studies about whether kinetic markers of embryo development can predict aneuploidy [35, 36]. The complexity of the matter of how aneuploidy and aberrant division patterns are intertwined is also investigated in the study of Chavez et al. [37], which stresses that embryo fragmentation might be a response to aneuploidy. It is interesting to observe that the same aberrant cleavage types as direct cleavage in three blastomeres of equal size are very frequent in triploid zygotes after conventional IVF. An extra pair of centrioles is not always the explanation for altered cleavage patterns in human tripronuclear embryos [38]. With implementation of time-lapse microscopy in IVF laboratories, along with recent advances in genetic research, we are gaining more and more knowledge and new perspectives on early human embryo development. We hope that future studies will give us more detailed information and it is certainly very important to shed light on the very frequent aberrant development of the early human embryo.

Although timings of specific events in early embryo development seem very promising indicators of their potential to form blastocysts, implant, and result in pregnancy, there are many indications that these morphokinetic markers cannot be used universally. They depend on many factors that influence embryo development. Kirkegaard et al. demonstrated that up to 31% of variation in timing of the events in embryo development can be explained by the origin of the embryo, including the patient-related and treatment-related factors [39]. Because of these limitations, as well as the small study size, timing of the events was not the primary study focus, but, rather, the occurrence of the cleavage abnormalities was. Our study showed statistically significant differences in the duration of the pronuclei, the duration of the first cytokinesis, the duration of the second cell cycle, and the synchronization of the cell division between 3- and 4-cell stages for the two study groups compared.

Time-lapse microscopy of embryo development in natural cycle in patients with POR gives us more data on embryo quality and implantation potential and consequently helps in counselling these patients about their chances to get pregnant. The population of poor responder patients is very heterogeneous [40]. It is therefore reasonable to expect that there are many different factors that influence their fertility and predicting success rates of the procedure. Knowledge of the factors that have an impact on embryo development and implantation potential is very important for counselling this group of patients. The process of how to present them with their chances for pregnancy and when to advise them to insist with stimulated cycle or switch to nonstimulated cycle or options such as donated oocytes or adoption is a delicate one.

It can be hypothesized that morphologically normal embryos with documented good dynamic in development are able to produce pregnancy in fair percentage even in poor responder patients. This fact can be used as additional information for counselling poor responder patients about their expectations. Large studies of abnormalities on embryos from nonstimulated cycles detected by time-lapse microscopy and their prevalence among various group of patients, different protocols, and patients ages would be needed to confirm our observations.

## 5. Conclusions

Abnormalities in early embryo development are frequent and the chances of transferring an embryo with no potential to implant are very high in the group of poor responder patients. Time-lapse microscopy of embryo development in poor responder patients successfully identified the group of embryos that had good potential to implant and those with irregular cleavage patterns that were very unlikely to implant. This data is very important for presenting these patients with their chances of success.

## Figures and Tables

**Table 1:** Comparison of the baseline patient characteristics and outcomes of transfers of normally and irregularly cleaved embryos.

	Cycles with transfer of normally developing embryo	Cycles with transfer of embryo with single or multiple cleavage irregularities	*P*
Number of cycles	26	27	
Age of women (mean, SD)	39.2 ± 3.0	39.0 ± 3.1	0.857
AMH (mean, SD)	0.35 ± 0.43	0.35 ± 0.28	0.594
Body mass index (mean, SD)	22.2 ± 3.0	23.5 ± 3.7	0.173
Previous treatment cycles (mean, SD)	3.3 ± 1.9	2.4 ± 2.1	0.019
Combined male & female infertility	15 (57.7)	13 (48.2)	0.586
Estradiol (mean, SD)	0.50 ± 0.32	0.40 ± 0.31	0.522
Biochemical pregnancy rate	8 (30.8)	0 (0)	0.002
Implantation rate	8 (30.8)	0 (0)	0.002
Clinical pregnancy rate	8 (30.8)	0 (0)	0.002
Live birth rate	6 (23.1)	0 (0)	0.010

Note: values in parentheses are percentages. AMH: anti-Müllerian hormone.

**Table 2:** Prevalence of different types of cleavage irregularities, relationship with classical morphology score at the time of transfer, and live births in 53 natural cycles of poor responders.

	Frequency	Good quality embryo (at the time of transfer) *N* (%)	Live births
Normally developing embryos	26 (49.1)	16 (30.2)	6
Embryos with single irregularity	16 (30.2)	2 (3.8)	0
Direct division in >2 blastomeres	7 (13.2)	2 (3.8)	0
Chaotic cleavage	1 (1.9)	0 (0)	0
Reverse cleavage	0 (0)	0 (0)	0
Developmental arrest	8 (15.1)	0 (0)	0
Embryos with multiple irregularities	11 (20.8)	0 (0)	0

Total	53	18 (34,0)	6

Note: values in parentheses are percentages.

**Table 3:** Comparison of timing of the events in early embryo development between normally developing embryos and embryos with cleavage irregularities.

Morphokinetic parameters	Normally developing embryos	Embryos with cleavage irregularities	*P*
	26	27	
*t*PB2	258.5 (90.5)	208.9 (88.7)	0.071
*t*PNa	608.6 (214.8)	556.0 (174.9)	0.359
*T*PNf	1460.9 (243.1)	1680.8 (526.7)	0.075
VP (*t*PNf − *t*PNa)	856.2 (184.8)	1014.3 (310.3)	0.037
ECC1 (*t*_2_ − *t*PB2)	1334.1 (143,3)	1610.9 (570.4)	0.096
*t*_2_	1624.2 (225.2)	1813.7 (497.9)	0.093
*t*_3_	2315.4 (211.4)	2472.4 (519.9)	0.256
ECC2	788.3 (173.7)	1064.6 (263.1)	0.005
Dck1	38.4 (42.9)	324.5 (656.5)	0.032
S2 (*t*_4_ − *t*_3_)	91.5 (152.3)	338.4 (361.2)	0.015

Note: mean time values are in minutes, SD in parentheses. *t*PB2: time passed from ICSI to the extrusion of the second polar body; *t*PNa: time from ICSI to the appearance of the both pronuclei; *t*PNf: time from ICSI to the disappearance of the pronuclei; VP: time period until the pronuclei become visible; ECC1 = *t*_2_ −  *t*PB2: the duration of the first cell cycle; *t*_2_, *t*_3_, *t*_4_: time from ICSI to the first frame in which two, three, and four cells are visible; ECC2 = *t*_4_ − *t*_2_: the duration of the second embryo cell cycle; dck1: the duration of the first cytokinesis; s2 = *t*_4_ − *t*_2_: the synchronization of the cell divisions between the 2- and 4-cell stages.
